# Successful Perioperative Management with Damage Control Surgery in a Patient with Massive Postpartum Hemorrhage of More Than 20,000 mL

**DOI:** 10.1155/2020/8872925

**Published:** 2020-06-16

**Authors:** Keisuke Yoshida, Kazuhiro Watanabe, Yuki Sato, Miho Sumiyoshi, Masahiro Murakawa

**Affiliations:** ^1^Department of Anesthesiology, Aidu Chuo Hospital, 1-1, Tsuruga-machi, Aizuwakmatsu, Fukushima 965-8611, Japan; ^2^Department of Anesthesiology, Fukushima Medical University, 1, Hikariga-oka, Fukushima 960-1297, Japan

## Abstract

Disseminated intravascular coagulation (DIC) in obstetrics is related to postpartum hemorrhage and has been a leading cause of maternal death. We here report a successful treatment, via damage control surgery (DCS), of a life-threatening massive hemorrhage of more than 20,000 mL due to DIC. A 30-year-old female was admitted to our hospital because of atonic bleeding. Since she was having a uterine rupture, an emergency hysterectomy was performed. Because of the severe DIC (fibrinogen, 65 mg/dL; platelet count, 6.0 × 10^9^/L), oozing persisted after the hysterectomy; thus, intraperitoneal gauze packing was performed as DCS. Afterwards, the coagulopathy was corrected, and the gauze was removed on the second postoperative day (POD 2). The patient was discharged without complications on POD 16. The present case demonstrated that performing DCS and waiting for improvement of the coagulation system can be one of the treatment options for management of patients with severe DIC.

## 1. Introduction

In obstetrics, disseminated intravascular coagulation (DIC) is associated with maternal deaths up to 25% in the world [1]. Transfusion and coagulation management are main treatment strategies in DIC and postpartum hemorrhage (PPH) [2]; however, some cases cannot be coped with by nonsurgical treatment. In many guidelines on PPH, hysterectomy is described as a last resort treatment strategy for severe PPH [3, 4], but damage control surgery (DCS) has not been described specifically. Although DCS is a common life-saving management in trauma [5], reports on DCS for severe obstetric hemorrhage such as our patient are lacking.

## 2. Case Presentation

A 30-year-old Japanese woman (height, 153 cm; weight, 45 kg), gravida 5, para 2, delivered a healthy infant by vaginal delivery at an obstetric clinic. She had no significant past medical history, and there was no problem during the course of her pregnancy. After the delivery of the infant and placenta, the bleeding could not be controlled, and the bleeding volume reached 2,000 mL. The patient diagnosed with PPH due to atonic bleeding with cervical laceration was admitted to our hospital after suturing of the cervical laceration and administration of crystalloid. At the clinic, blood transfusion was not performed.

On arrival at our hospital, although anemia was existing (hemoglobin concentration, 6.3 g/dL; hematocrit, 18.4%), the results of the blood coagulation test were almost within normal limits except for D-dimer (platelet count, 135 × 10^9^/L; prothrombin time-international normalized ratio (PT-INR), 0.92; activated partial thromboplastin time (APTT), 30.5 sec; fibrinogen, 258 mg/dL; D-dimer, 7.5 *µ*g/mL). The bleeding could not be controlled by uterotonics, intrauterine balloon tamponade, or blood transfusion as replenishment of coagulation factors. She developed hemorrhagic shock, which leads to an emergency hysterectomy under general anesthesia.

At the beginning of the surgery, she was acidotic (arterial blood pH, 6.97) and hypothermic (body temperature, 33.8°C). Intraoperatively, uterine rupture due to cervical laceration followed by DIC (fibrinogen, 65 mg/dL; platelet count, 6.0 × 10^9^/L at an intraoperative blood test) was diagnosed based on the obstetrical DIC score, which is commonly used in Japan [6, 7]. After the hysterectomy, oozing related to DIC persisted; therefore, gauze packing was inserted into the pouch of Douglas as DCS by the obstetricians, and temporary abdominal closure was performed. During the surgery, her mean arterial pressure was maintained within 60–75 mmHg and urine output was maintained at >1 mL/kg/hr. After the surgery, the patient was transferred to the intensive care unit (ICU) with mechanical ventilation.

On the day of surgery, blood transfusion was continued and the patient recovered from hemorrhagic shock. In addition, 4.5 g tazobactam/piperacillin was administered intravenously every 8 hours while packing was in place. On the first postoperative day (POD 1), the coagulation status gradually improved. A surgery to remove the intraperitoneal gauze was done on POD 2 because the bleeding from the drains had been decreased. Although there was a subcutaneous hematoma, bleeding in the peritoneal cavity had stopped. The total cumulative bleeding volume at POD 2 was 23,929 mL.

**Figure 1:**
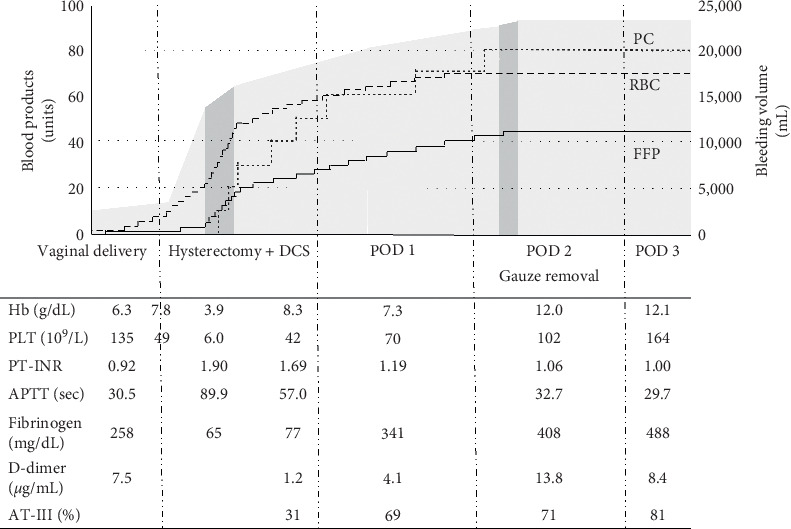
Clinical course and blood test results.

After the second surgery, the patient's general condition was gradually improved. The artificial respiration was ended on POD 5, and she was discharged from the hospital without complications on POD 16. Her clinical course and blood test results are shown in Figure 1.

## 3. Discussion

The present case was a successful treatment of life-threatening massive PPH due to DIC. The reported definitions of DIC are varied, meaning that several definitions and guidelines are currently used. Many define DIC according to its clinical characteristics using various scoring systems [1]. Although there is controversy regarding dealing with coagulopathy related to massive hemorrhage, pure obstetric hemorrhage is one of the causes of DIC [1], which also worsens PPH; thus, the scoring system is reasonable for recognizing DIC/coagulopathy related to massive hemorrhage. In the present case, we made the diagnosis of DIC based on the patient's obstetrical DIC score [6, 7] which evaluates underlying diseases (e.g., placental abruption, amniotic fluid embolism, and postpartum hemorrhage), clinical symptoms (e.g., organ failure, hemorrhage diathesis, and shock symptoms), and laboratory findings.

Although there are a few reports of DCS applied in obstetrics, Pacheco et al. [8] described that several obstetric conditions may benefit from DCS. According to the previous report analyzing the 53 DCS cases performed for PPH by Deffieux et al. [9], this case is classified into the DCS failure group in terms of the amount of RBC used, and the mortality rate is considered to be 65%. Additionally, this case was more severe compared with some previous case reports of DCS for PPH [10, 11]. In the present case, it was extremely difficult to obtain sufficient hemostasis during hysterectomy because her coagulation system was collapsed at the time. Therefore, the strategy of DCS and waiting for improvement in her general condition was reasonable. As a result, reoperation (gauze removal) was performed at POD 2 because the patient's body temperature, blood pH, electrolyte anomalies, and coagulopathy had been corrected [8]. In addition, the present case had several strengths which contributed to the positive outcome. Firstly, many blood products were able to be used intensively in the acute phase, and there was no complication caused by massive blood transfusion such as cardiorespiratory adverse effects including transfusion-associated circulatory overload [12] and transfusion-related acute lung injury [13]. Secondly, the present case was rapidly transported to our general hospital from a primary clinic. Our hospital has an emergency and critical care center; therefore, there is a lot of experience of DCS management for abdominal trauma.

Meanwhile, there are some points to reflect upon. In multiple guidelines and reviews on obstetric hemorrhage management [2, 3, 14, 15], the importance of fibrinogen as a coagulation factor is emphasized. They recommend administering red blood cell (RBC) and fresh frozen plasma (FFP) in a ratio driven protocol. However, the blood transfusions performed at an early stage in this patient had a higher ratio of RBC to FFP; that is, starting administration of FFP was too late. The reason was it took time for transportation of FFP from the blood center to our hospital and thawing them. This result may have led to an increase in bleeding volume and consequent deterioration of DIC.

Regarding platelet transfusion in the PPH setting, it is recommended that platelet concentrates (PC) should be administered if the platelet count is <50 × 10^9^/L [14, 15]. However, because of time taken for delivery, the number of platelets in our patient had already dramatically decreased at the time platelets arrived at our hospital. Since DIC often develops rapidly, it is necessary to properly anticipate changes in the patient's condition and to order blood products at an early stage.

There was also an option to try interventional radiology before hysterectomy, such as uterine artery embolization (UAE). While there is a possibility that UAE can retain fertility, 15% of cases require hysterectomy after UAE [16]. In the present case, we selected emergency hysterectomy based on the serious condition of the patient and difficulty of emergency UAE at nighttime in our hospital.

Fibrinogen of 150–200 mg/dL is too low for adequate hemostasis during ongoing PPH. Several reports have suggested the usefulness of fibrinogen concentrates for PPH [2, 4, 14, 17]. After this experience, fibrinogen concentrates were adopted in our hospital, although their use for PPH and DIC is not licensed in Japan. We will consider using them after obtaining informed consent when encountering similar cases in the future.

Figure 1 shows the accumulated bleeding volume (stained with gray), the accumulated amount of blood products used (three lines), and the results of blood tests from the day the patient was transferred to our hospital until POD 3. The dark gray stained ranges represent the durations of the surgeries. Blood products used in Japan are as follows: 2 units of RBC (approximately 280 mL) contain red blood cells derived from 400 mL of blood. 2 units of FFP (approx. 240 mL) contain fresh frozen plasma derived from 400 mL of blood. 10 units of PC (approx. 200 mL) contain platelets more than 2.0 × 10^11^ (RBC, red blood cell; FFP, fresh frozen plasma; PC, platelet concentrates; DCS, damage control surgery; POD, postoperative day; Hb, hemoglobin concentration; PLT, platelet count; PT-INR, prothrombin time-international normalized ratio; APTT, activated partial thromboplastin time; AT-III, antithrombin-III; BT, body temperature).

## 4. Conclusion

The present case demonstrated that waiting for improvement of the coagulation system by DCS is a possible treatment option for severe PPH and obstetrical DIC.

## Data Availability

The data that support the findings of this case report are available on request from the corresponding author, KY. The data are not publicly available due to their containing information that could compromise the privacy of the patient.

## Abbreviations

DIC: Disseminated intravascular coagulation
PPH: Postpartum hemorrhage
DCS: Damage control surgery
PT-INR: Prothrombin time-international normalized ratio
APTT: Activated partial thromboplastin time
ICU: Intensive care unit
POD: Postoperative day
RBC: Red blood cell
FFP: Fresh frozen plasma
PC: Platelet concentrates
UAE: Uterine artery embolization.
